# Anæsthesia

**Published:** 1848-03

**Authors:** 


### THE MEDICAL EXAMINER.

PHILADELPHIA, MARCH, 1848.

### ANÆSTHESIA.

When our Boston brethren first announced the discovery of an agent for producing complete insensibility during surgical operations, we expressed our candid, but very serious doubts, whether the condition of the nervous system could be so modified without imminent danger. Our fears on this point are not yet entirely removed; but, as intimated in our last number, the cases in which the agents have been employed for this purpose, are now so numerous, and the aggregate results so favourable, that we cannot deny that the dangers from their employment seem to be far less than we apprehended.

The dawn of this great discovery—the greatest, perhaps, of the age—was singularly infelicitous in its attending circumstances. The murky clouds of avarice hung thickly about it, and shut out generous confidence and hope, by the suspicions and reasonable doubts which such unworthy aims naturally excited. So long as cupidity sought to conceal the agent under a strange name, and to limit its usefulness by the penalties of a patent, we felt that we could have neither lot nor part in it:—we were unalterably determined to let it alone. Now that the empirical garments with which its fair proportions were veiled and deformed, have been stripped off, and every eye may view it without prejudice, we may contemplate the subject with the calmness which becomes its deep importance. To our country belongs, in part, the honour, not only of the discovery of the now most popular agent, chloroform, but of the much greater discovery of the powers of the ether, which has led to the employment of both as anæsthetic agents; and but for the meretricious character first cast upon this noble contribution to science and humanity, how proudly might Americans point to it and claim it as their own! However, the subject is now open to the widest range of experiment and observation. While it is conceded, that both sulphuric ether and chloroform, will, by inhalation in sufficient quantity, produce insensibility to the most painful lesions, it remains to be determined which is the best adapted for general use, as well as the extent to which the influence of the agent may be safely carried; the circumstances most proper for its employment, and those which render it hazardous or altogether improper. Time and observation alone can give us this information, and prudence forbids that we should be in haste in forming our conclusions. The communications, contained in our present number in relation to these points, are of great value. It will be seen that Professor Simpson is unqualified in his preference for chloroform, and that he deems it quite a safe remedy, even although he admits that symptoms are often induced that must alarm a novice. Professor Meigs, without denying the utility of this means of allaying pain as a pathological or morbid symptom, contends that the ordinary pains of labour are natural or physiological, and therefore not to be interfered with. This is a very important distinction, and our prepossessions, at least, coincide with the opinions of our colleague on this point.

The cases reported by Dr. Clarke, in which sulphuric ether was employed to alleviate the pains of labour, seem almost conclusive as to the utility and safety of the remedy. We have nowhere seen more decided or satisfactory results,—the more satisfactory to us, indeed, because of our knowledge of the plain, practical, and altogether truthful character of the reporter.

The last quarterly report of the College of Physicians of Philadelphia contains a report on this subject, by Dr. Isaac Parrish, embracing an excellent summary of the cases published prior to that time, from which the reporter arrives at conclusions very similar to those we have expressed in the course of these remarks.

